# Software for the care of people with cardiovascular risk: construction and evidence of validity

**DOI:** 10.1590/0034-7167-2024-0276

**Published:** 2024-12-16

**Authors:** Nuno Damácio de Carvalho Félix, Raércia dos Santos Carneiro, Luis Filippe Rasia Pacheco, Brenda Silva Cunha, João Cruz, Johann Aires Boness, Mariana Carvalho Gavazza, Alba Lúcia Bottura Leite de Barros

**Affiliations:** I Universidade Federal do Recôncavo da Bahia. Santo Antônio de Jesus, Bahia, Brazil; II Universidade Federal do Ceará. Fortaleza, Ceará, Brazil; III Secretaria Municipal de Saúde. Salvador, Bahia, Brazil; IV Universidade Federal de São Paulo. São Paulo, São Paulo, Brazil

**Keywords:** Software, Software Validation, Heart Disease Risk Factors, Nursing Theory, Primary Health Care., Programas Informáticos, Validación de Programas de Computación, Factores de Riesgo de Enfermedad Cardiaca, Teoría de Enfermería, Atención Primaria de Salud.

## Abstract

**Objectives:**

to build and validate software for the care of people with cardiovascular risk.

**Methods:**

a methodological study, applied to software development, anchored in a nursing theory and classification system, in three stages: 1) requirements engineering; 2) software architecture and coding; and 3) testing and content validity by 12 experts in computer science, with a Content Validity Ratio score.

**Results:**

called e-TEORISC, in software format, for nursing care for people with cardiovascular risk, online and offline, containing a database linked to the Nursing Process stages. Experts considered that the attributes of functional suitability, performance efficiency, reliability, maintainability, usability, safety and portability obtained desirable scores.

**Conclusions:**

e-TEORISC has evidence of validity to instrumentalize care for people at cardiovascular risk, with potential for technology transfer to the Brazilian Health System.

## RESUMEN

**Objetivos:**

construir y validar *software* para la atención de personas con riesgo cardiovascular.

**Métodos:**

estudio metodológico, aplicado al desarrollo de *software*, anclado en una teoría y sistema de clasificación de enfermería, en tres etapas: 1) ingeniería de requisitos; 2) arquitectura y codificación de *software*; y 3) pruebas y validación de contenido por parte de 12 expertos en el campo de la informática, con un puntaje de Ratio de Validez del Contenido.

**Resultados:**

denominado e-TEORISC, en formato *software*, para la atención de enfermería a personas con riesgo cardiovascular, *online* y *offline*, que contiene una base de datos vinculada a las etapas del Proceso de Enfermería. Los expertos consideraron que los atributos de adecuación funcional, eficiencia de desempeño, confiabilidad, mantenibilidad, usabilidad, seguridad y portabilidad obtuvieron puntuaciones deseables.

**Conclusiones:**

e-TEORISC tiene evidencia de validez para brindar atención a personas en riesgo cardiovascular, con potencial de transferencia tecnológica al Sistema Único de Salud.

## INTRODUCTION

Technologies have a direct impact on people’s lives by assisting in care practices and lifestyles. The process of globalization and the rapid development and availability of technological products increase access to information and determine integration/interaction in the provision of healthcare services^(1)^, including in this area the context of cardiovascular risk, which requires technical and technological production to meet a population’s real needs.

Cardiovascular risk comprises a health and care context dependent on the identification of modifiable and non-modifiable risk factors that directly impact the potentialization of acute and/or chronic conditions that affect the circulatory system^(2)^, thus representing an emerging demand for nursing care. Therefore, it is part of the scope of nursing science to develop and assess available evidence on strategies for controlling and preventing cardiovascular risk through technologies. There is an emerging need to advance knowledge production and foster discussions on the development of technologies with ethical and social commitment and with a view to solving problems in nursing care practice and sustainability.

New technologies have been the object of study in nursing, with a view to resolving emerging demands in everyday life, guided by a theory to transcend thinking and acting to analyze, interpret and intervene in the practical process^(3)^. Among the various types, assistive technologies comprise the result of the application of methods and techniques that increase the possibilities of care in response to people’s needs, with impacts on the way care is provided and the responses obtained^(4)^.

In nursing, the validity of assistive technologies has been developed to aid practice, citing software, in particular, as innovative tools based on computerized language that guided nursing interventions, with objectives such as contemplating an interactive and incremental process, operationalizing the logistical-operational system, reducing the time to implement activities and facilitating the identification of care demands through continuous reassessment^(5,6)^. These are essential objectives for the development of assistive technologies with strong evidence of validity.

For use in this study, we chose to define assistive technologies with a focus on evidence of validity, for the development of clinical skills in nursing and healthcare professionals in the care of cardiovascular risk, especially in Primary Health Care. Technology is a domain interrelated to nursing, with reference to the metaparadigm, and can provide positive effects on the construction of knowledge^(7)^, with potential contribution to the reduction of cardiovascular risk factors in the population and technology transfer to the Brazilian Health System (SUS - *Sistema Único de Saúde*).

There is strong evidence of the effectiveness of personalized nursing interventions on the quality of life of people with heart disease^(8)^, and the results are promising when nursing care for these people is combined with the development of assistive technologies^(9)^, especially when it is based on a theory and uses a nursing classification system.

Standardized language systems assist nurses in obtaining responses to a clinical risk situation or illness process to which individuals are subject. In this regard, there is a lack of verification of the needs of different populations through access to a well-structured care plan containing nursing diagnoses, especially in primary care, a scenario that still lacks structured processes guided by nursing classification^(10)^. Furthermore, documenting the Nursing Process (NP) is one of the ethical prerogatives of nursing care, increasing the quality and integration of care through five well-established, interrelated, recurrent, interdependent and cyclical stages, mapping, in a hierarchical way, different ways of using language systems^(11)^.

Therefore, it is necessary and emerging in the context of nursing practice a technology capable of helping nurses to operationalize care plans for patients at cardiovascular risk, reducing consultation time, directing care proposals and enhancing practice indicators. The software built and assessed in this study is presented as original and innovative, as it promotes advancement in knowledge, combining nursing science with technological proposals in the decision-making process led by nursing professionals for the care of people in the context of cardiovascular risk, in which there is a shortage of technologies like this on the international scene.

## OBJECTIVES

To build and validate software for the care of people with cardiovascular risk.

## METHODS

### Ethical aspects

The research was approved by the *Universidade Federal do Recôncavo da Bahia* Research Ethics Committee, and complied with the ethical precepts of Resolution466 of 2012 of the Brazilian National Health Council.

### Study design

This is a methodological research applied to technological development^(12)^, of the software type, based on *Teoria do Cuidado no Contexto de Risco Cardiovascular* (TEORISC, Theory of Care in the Context of Cardiovascular Risk)^(13)^ assumptions and productions associated with the use of the International Classification for Nursing Practice (ICNP^®^) in relation to the phenomena of cardiovascular risk and metabolic syndrome^(14-17)^.

### Methodological procedures

Using the software engineering technique^(18)^, three development stages emerged: 1) requirements engineering; 2) software architecture and coding; and 3) content testing and validity by experts.

### Stage 1 - Requirements engineering

It consists of applying specialized techniques to group functionalities and describe them with a view to consistent implementation. The tasks are: review and clarification of user demands; distribution, documentation and review of requirements by users. The requirements were recorded in user data to facilitate communication between the technical team and the members of the Cardiovascular Care Research and Extension Group (GPCARDIO).

To cover this stage, five phases were carried out (SI - Kick-Off; SII/III - product vision; SIV - product Is - Is not - Does - Does not; SV - product objectives; and SVI - personas). The second phase was divided into two moments (SII and SIII). The meetings were conducted with the help of a programmer, virtually, using the Lean Inception method, in order to define a Minimum Viable Product (MVP) or prototype to then build the software.

The SI “Kick-Off” consists of the presentation and explanation of the method used and its developers. The SII/III “product vision” is used to chart the initial path of a product. In SIV, the product objectives are discussed. SV corresponded to the development phase of the personas, identifying potential technology users through the creation and characterization of fictitious characters.

The Lean Inception method aims to accelerate the primary stage of product development and discover the most relevant points for creating a software, making it objective. Moreover, it promotes MVP understanding, which corresponds to the simplest version that will be presented to users. Furthermore, MVP can guide developers on software usefulness as well as understand the user profile and how a product can add value and help them^(19)^.

MVP was built to identify the profile of users with cardiovascular risk as well as the most relevant points for strategies that determine solutions to reduce the risk of acquiring cardiovascular diseases. GPCARDIO members worked on the software construction phases in discussions with an engineer about the necessary requirements that should be contained in the technology, concepts, structure, modeling, tab sequencing and construction of hypothetical cases.

### Stage 2 - Software architecture and coding

It involves defining a system structure and behavior that meets users’ expectations in terms of efficiency, consistency, and security, for instance. To develop software architecture, three main tasks were performed: a) identification of quality attributes with description of architectural scenarios; b) making architectural decisions; and c) elaboration of architectural visions for development^(18)^.

In task A, the screens were developed using the Web Canva^®^ software, with subsequent organization, formatting and structuring by the programmer responsible for developing the software. The defined architecture ensures system quality and its documentation, assisting developers during coding. In task B, regarding software information security, access to the data was only permitted to users with a specific registration number linked to the research, previously registered by the administrator - researcher proposing the study. The software was installed on a network server with encrypted passwords, in order to increase entry security and registration in the system.

In task C, for software development, coding and testing were performed by a programmer. The programming language used was React with Typescript, to create the frontEnd. SASS was used for graphic styling, and Vercel was used for the hosting platform. MSQL was used as the database management system, which uses the Node.js language, with TypeScript as the interface, and is hosted on the Railway company’s cloud server.

The concepts “cardiovascular risk”^(2)^ and “metabolic syndrome”^(17)^ were used to develop the software, since the syndrome brings together important cardiovascular risk factors, in which the main precautions should be considered in technology construction and validity.

Finally, as the software’s main content tool, a terminological subset of ICNP^®^ was used, with 169 nursing interventions, 55 diagnoses and 36 outcomes divided into cardiometabolic, behavioral, psychosocial and cultural, work, emotional and therapeutic factors^(13)^. It is worth noting that the subset was validated by 88 experts and applied to 22 patients with metabolic syndrome^(13)^. Thus, the instrument was operationalized in software format, maximizing the possibilities of classification system use, application and evolution.

### Stage 3 - Content testing and validity

The Delphi technique was chosen for testing and validating the software content, carried out by a committee of anonymous experts^(20)^ to analyze item representativeness^(21)^, totaling 12 computer science experts. The International Organization for Standardization (ISO) and the International Electrotechnical Commission (IEC) standards, specific for software assessment, were used as a basis.

The first stage of the ISO/IEC 25040 Assessment Process is the ISO/IEC 25010 Product Quality Model, in which eight characteristics and 31 sub-characteristics aimed at information technology experts are assessed, namely: functional suitability (functional completeness, functional correctness and functional appropriateness); performance efficiency (time, resources and capacity); compatibility (co-existence and interoperability); usability (recognition of adequacy, learnability, error protection, operability, user interface aesthetics and accessibility); reliability (maturity, fault tolerance, recoverability and availability); security (confidentiality, integrity, non-repudiation, accountability and authenticity); maintainability (analysability, modifiability, modularity, reusability, testability); and portability (adaptability, installability and replaceability)^(22,23)^.

Validity was performed by 12 experts, identified via the *Lattes* Platform, according to inclusion criteria, such as being professionals in information technology, systems development or computer science, with experience in software creation and computerization in healthcare or software validity and adaptation. The expert who met all the requirements was included in the research. After prior contact via email, the Informed Consent Form, a summary presentation protocol of the research project, the adapted instrument and instructions for completing and assessing the items to be analyzed were sent, in addition to a link to access the software. A period of 20 days was stipulated to return the analyzed material.

The Content Validity Ratio (CVR) was used, in which experts assessed each instrument item as +1 (adequate), -1 (adequate with changes) or 0 (inadequate). These measure item consistency, with those that obtain a minimum percentage of 80% being considered relevant^(24)^.

### Data analysis

The results of the first two stages were structured and analyzed according to the Ruby, Ruby on Rails and JavaScript programming languages, using the Bootstrap framework and the implementation of a production server, where Ubuntu Linux and Nginx Webserver were executed. Content testing and validity processes through RCV were structured using descriptive statistics.

## RESULTS

The research team, composed of five nursing students, such as three nursing professors, nurse researchers and a software engineer, participated in the requirements engineering phase. In the SI phase, the presentation/explanation of the method and its developers were structured. SII phase helped to pave the initial path of the product, with definitions for whom the technology is intended (adults), what problem it would solve (inadequate care for cardiovascular risk), product name (e-TEORISC) and what the product will be (application software).

Phase SIII defined the benefit that e-TEORISC brings to the problem (preventing cardiovascular diseases in the medium and long term), the competition (traditional clinic) and the advantage (e-TEORISC based on a theory and a nursing classification system). It is important to note that e-TEORISC is primarily aimed at people with cardiovascular risk factors (without disease). Concerning the differential of e-TEORISC, it was identified that it will be able to integrate healthcare professionals to identify and assist, in an efficient and effective manner, the cardiovascular risk of people.

In the SIV phase, it was understood that e-TEORISC is an application software, a tool for healthcare professionals led by nurses and organized by the multidisciplinary team, which will operate online and offline, containing a database. This database is where patient registration information, care plans and goals shared among professionals will be stored. It was defined that e-TEORISC will not be a social network nor will it be an automatic assistant, website, checklist or protocol.

As for what e-TEORISC does, it was defined that the software guides care for cardiovascular risk, monitors clinical and laboratory parameters, clarifies doubts, shares instructional material, promotes self-care, provides guidance on work comfort measures and assesses quality of life. As for what e-TEORISC does not do, the software will not determine diagnoses/outcomes/interventions or therapeutic plans, maintaining professionals’ autonomy in clinical reasoning to be guided by the software.

In the SV phase, e-TEORISC aimed at: identifying cardiovascular risk and metabolic syndrome; equipping health and nursing professionals with the necessary tools for care in the context of cardiovascular risk; assisting in prescribing actions to reduce cardiovascular risk in a systematic manner; promoting the active participation of a person in the care of cardiovascular risk factors; and having interoperability for possible technological transfer to the SUS. In SVI, cases of three characters were constructed, such as a nurse, a community health worker and a nurse, defining their sociodemographic and behavioral characteristics and health needs to observe a system testing.

In terms of software architecture and coding, e-TEORISC construction was developed through a technology structure divided into an initial section with videos, the e-TEORISC logo and an access button. The videos cover how to use the software, how it works and how to access the available resources (optional viewing), with information about the platform’s creation team, with photos, names and training of these people.

**Figure 1:**
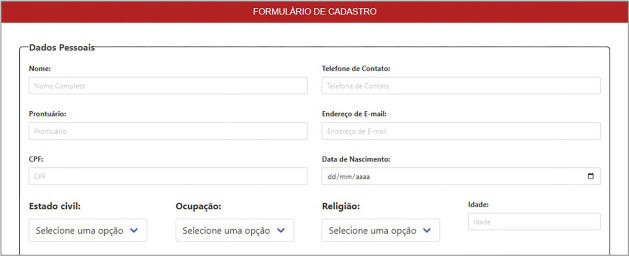
Statement of a person’s registration in e-TEORISC, Santo Antônio de Jesus, Bahia, Brazil, 2023

It is important to note that the technology is for management purposes by nurses and for monitoring by the healthcare team, as patients themselves can register for monitoring by the team. On the home screen, the access button will show three options: healthcare client (person); healthcare professional (care provider); and developer (software controller). On the first access, healthcare clients will register in person or by self-completion, requiring full name, ID card, age, race, social name and sex. At the end, clients must express agreement with the terms of use (Figure 1).

The initial in-person consultation must be with a nursing or healthcare professional, which can be scheduled via the software. Clinical and anthropometric data will be provided, as well as questions about physical exercise, sleep and rest, support network, personal and/or family comorbidities, and beliefs. To facilitate information distribution, the screens were divided according to the set of TEORISC risk factors and the information for filling out, tracking, and structuring care plan through the ICNP® subset.

**Figure 2:**
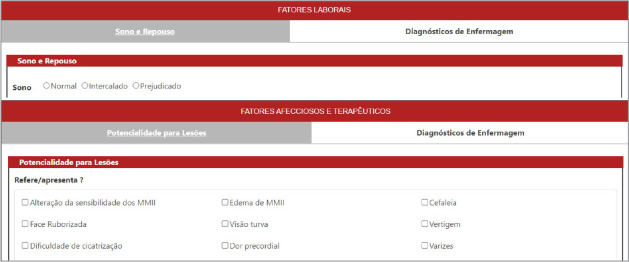
Demonstration of data related to the Nursing Process in e-TEORISC, Santo Antônio de Jesus, Bahia, Brazil, 2023

**Figure 3:**
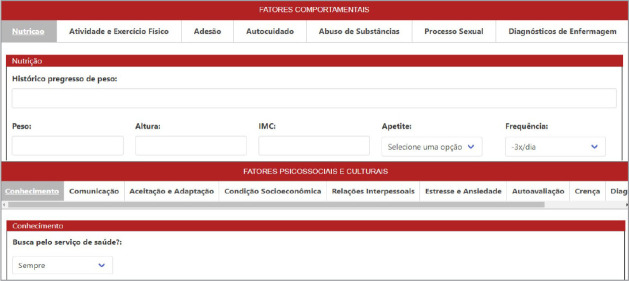
Demonstration of data related to cardiovascular risk factors in e-TEORISC, Santo Antônio de Jesus, Bahia, Brazil, 2023

Through e-TEORISC, nurses can search for a person’s name and then document NP, through nursing assessment, diagnosis, planning, implementation and evolution, making the results available in the “records” tab and being able to access the records of other professionals, as observed in Figures 2 and 3.

After the first consultation, healthcare clients will be able to access their history of consultations. Other healthcare professionals will be able to participate in interconsultations (if necessary), and with scheduled dates and times. Records will be made according to each professional’s individualized care plan, with access to other professional records and client data. Developers will have access to all information on the platform, including the database (in the cloud) after registration.

Expert evaluators have a background in computer science (100%), including doctoral degree (40%), master’s degree (50%) and specialization (10%), and their professional activity is predominantly in teaching (60%), followed by autonomous activities (40%) related to the development and assessment of technological products, such as software, for healthcare services. In content tests and validity, e-TEORISC obtained an average of 89.9% of the CVR, considering the content valid according to Table 1. Among the attributes, portability stands out with CVR of 100%. It is noteworthy that usability, security and portability obtained scores above 90%. Functional suitability, performance efficiency, reliability and maintainability presented sub-characteristics with percentages of 80% to 85%.

**Table 1:** Percentage of agreement of experts regarding the Content Validity Ratio of each attribute and sub-characteristics in relation to the e-TEORISC software, Santo Antônio de Jesus, Bahia, Brazil, 2023

Attributes	Sub-characteristics	CVR (%)^*^
Functional suitability	Functional completeness	85
	Functional correctness	85
	Functional appropriateness	85
Eficiência de desempenho	Co-existence	80
	Interoperability	85
	Appropriateness recognizability	85
Compatibility	Learnability	75
	Error protection	75
Usability	Operability	95
	User interface aesthetics	100
	Accessibility	95
	Maturity	95
	Fault tolerance	100
	Recoverability	95
Reliability	Availability	75
	Confidentiality	85
	Integrity	85
	Non-repudiation	85
Security	Accountability	95
	Authenticity	90
	Analysability	90
	Modifiability	90
	Modularity	100
Maintainability	Reusability	85
	Testability	85
	Adaptability	80
	Installability	80
	Replaceability	85
Portability	Co-existence	100
	Interoperability	100
	Appropriateness recognizability	100

* *CVR - Content Validity Ratio.*

The subcategories co-existence, interoperability and maturity obtained CVR below the established (75%), in which experts judged that e-TEORISC needs to advance in relation to the software already existing in the SUS, as well as clinical testing, to strengthen the indicators related to the items. The main considerations are presented below: improve the layout structure of tools related to nursing consultation items; enhance functions more efficiently while sharing an environment or resources with other products; improve the software’s ability to enable two or more systems, products or components to exchange information; and improve the software’s ability to strengthen reliability, through assessment by primary care professionals and robust clinical tests, providing feedback and maturing e-TEORISC. As this is a technology institutionally linked to the assets of the university where it was developed, the software will be registered with the *Instituto Nacional da Propriedade Industrial* and, after the brand is granted to the university, it may be made available for technology transfer to the SUS.

## DISCUSSION

The e-TEORISC was designed based on NP and founded on TEORISC, which predicts the phenomenon of cardiovascular risk and assists in care practices with health promotion, which permeates a new level of empowerment for primary care nurses in the containment of injuries and prevention of diseases, in addition to allowing the generation of indicators based on specialized nursing language. Its use allows for the creation of a care program for people at cardiovascular risk, through the ability to screen, identify risks, propose interventions and assist with clinical nursing reasoning, specifically regarding cardiovascular health.

In research that implemented NP in hospital inpatient units, it was predicted that the use of software can enable decision-making, clinical judgment, provide diagnoses, results and nursing interventions in modus operandi and directive^(25)^. However, continuity of care and use of the system was not fully achieved, which leads to weaknesses in terms of technology transfer to the healthcare service. This is a crucial point in e-TEORISC operation, as it enables agility in NP and ensures effective applicability in services, and can demonstrate rapid absorption by nurses in the service.

In the field of cardiovascular health, there are software programs that respond to the needs of checking electrocardiogram curves as an aid in identifying anomalies related to cardiac pathologies, as well as the concept of heart failure, causes, signs and symptoms, diagnosis and treatment^(26,27)^. In the field of nursing care, no software was found to support nurses in carrying out care plans for patients with cardiovascular risk, which constitutes an important gap in the international scenario. In e-TEORISC, nurses are allowed to type observed responses not included in the screen layout presented, which significantly advances the development of the software and does not limit itself to a standard scope, enabling its continuous remodeling.

As for on-screen presentation, we can see the production of viable software, with typical structures of machine architecture and language (mechanical learning), which brings together five well-defined phases that enable the system to be operationalized. However, due to the novelty of a technological production in the area of cardiovascular risk, it is important to define its need for adaptation to the digital environment, as well as frequent updates specific to research with this scope.

The e-TEORISC was divided into groups of factors that make up the theory that underpinned this study, and was therefore concerned with grouping all indicators pertaining to cardiovascular risk with diagnoses and interventions grouped and segmented for each group of factors. It is understood that a system that encompasses the largest number of elements capable of identifying a nursing phenomenon ensures better results for professional dynamics^(28)^. From this perspective, TEORISC’s contribution to this study involves different aspects, focusing on NP stages through the instrument constructed and validated^(13)^, as a basis for understanding NP stages with the prevention of cardiovascular diseases, improving clinical assimilations of nursing professionals.

The software allows the identification of cardiovascular risk factors, outlining care alternatives based on clinical decision-making. In contrast, the literature describes the use of the REC24h software, which is part of the Study of Cardiovascular Risks in Adolescents, but limited to the factors of obesity, changes in lipid and glycemic metabolism and blood pressure^(29)^.

Software, in general, is already part of the routine of healthcare services, with a view to expanding a holistic, accessible and widely used method. In some cases, it helps to operationalize services with the purpose of bringing together a database to generate reports, flows and dictionaries^(30)^. In this case, the software in question uses ICNP® as a classification system. In addition to 55 nursing diagnoses, 169 interventions and 36 outcomes for the population at cardiovascular risk, nurses can choose which diagnoses and interventions they wish to apply to individuals, based on data grouping and decision-making. In this regard, a study points to the need to insert standardized languages in care technologies that promote guidance for NP, through decision-making support, in a way that is accessible to both professionals and users^(25)^. This prerogative was contemplated in the study in question.

The use of software implements logical and standardized languages in an attempt to structure care plans for cardiovascular risk. Thus, software with this theme can corroborate the tracking of cardiovascular risk, and tend to encourage self-care with health when this risk is identified. As a consequence, there is a reduction in morbidity and mortality due to cardiovascular causes^(31)^. In turn, attention should be paid to strategies already implemented to reduce the risk of developing heart disease in order to improve care strategies.

Software is a tool that makes it possible to optimize nursing records and, therefore, streamline their actions. In this way, it helps in decision-making by combining different sources of knowledge, such as software engineering and clinical reasoning, formulating appropriate user profiles^(25)^, direct and objective records of health information in different contexts^(26)^, freedom, standardization, reduction of time in bureaucratic records and facilitated retrieval of information^(32)^, in addition to the need for continuing reassessment^(33)^. It is emphasized that the e-TEORISC on screen has the purpose of corroborating the objectives set out in the literature, as well as the systematic storage of information.

The devices disseminated in the scientific literature do not predict or guide care, but focus on identifying risk. HEARTS is an initiative of the World Health Organization targeting Primary Healthcare that aims to improve the prevention and control of myocardial infarction, stroke or cardiovascular death over a ten-year period^(34)^. Another app is the ASCVD Risk Estimator, which estimates cardiovascular risk for the next ten years. It is also not an app that can guide a consultation, but rather works as a tracker for some risk factors, highlighting that the risk of developing cardiovascular diseases can only be calculated considering the age range of 40 to 79 years^(35)^. Although they are important tools, they do not include several risk factors necessary for a more complete assessment of the health of a person at cardiovascular risk.

In nurse-led care programs in cardiovascular health, interventions are based on the use of nursing theories that allow for increasing resilience in patients after coronary angioplasty^(36)^, or in reducing risk factors after an acute event, such as coronary syndromes^(37)^. In this case, it is essential that nurses assess e-TEORISC and propose alternatives to improve the system, aiming at its leadership in cardiovascular care and adaptation to their needs and different realities.

Studies with validity of software in nursing observed a similar proportion of validity to that found in the study in question, such as in the software for heart failure, with validity greater than 0.80 in its items^(26)^, and software for hemodialysis care (0.89)^(38)^, which supports the study in question. Most of categories assessed in this study received CVR greater than 0.80, except for co-existence, interoperability and maturity, indicating the demand for follow-up in e-TEORISC development and clinical assessment. These require revision, as in other studies that achieved scores lower than 0.78^(38)^. On the other hand, a study with software for preventing injuries in newborns, using the same ISO/IEC 25040 standard, adopted definitions above 0.70 as valid^(39)^. This alternative provides support for slight revisions in the judgments that obtained a score lower than 0.75 in the study in question.

Factors such as work and family stress, sedentary lifestyle and family history are absent in other technologies^(32)^, being another differential for assistive technology resulting from this study, which is based on a nursing theory in which the concept of cardiovascular risk itself was analyzed, giving robustness to the software. Similar studies also confirm the idea that assistive technologies, such as software, help to make healthcare services more agile in the stages of collecting, recording, storing and handling patient data, which helps to reduce possible errors in the care process^(33)^.

The construction of this technological product, based on TEORISC, provides theoretical support to the software, bringing elements that make it possible to contribute to nurses’ critical thinking in NP operationalization^(11)^. This process culminates in the translation of theoretical knowledge into practice. Furthermore, it is important to highlight the need for special attention to cardiovascular risk factors, since these are multiple and contribute to the increased risk of developing cardiovascular diseases, as well as the increased morbidity and mortality, and there is a need to recognize these factors in the process of developing care^(2)^.

In this way, TEORISC, associated with the developed software, is a valuable instrument for nursing practice, as it guides healthcare from nurses’ perspective, scientifically based and using a standardized language system, also contributing to the dissemination of the importance of using classification systems in nursing and the incorporation of NP into daily practice, which in turn can generate data and indicators for healthcare services.

### Study limitations

Experts’ suggestions have not yet been fully incorporated into e-TEORISC due to the procedures required to modify the system. The software needs improvements to be incorporated into the SUS with all its operational functions. The participation of members with expertise in the area and adherence to methodological rigor were followed to reduce the limitations.

### Contributions to nursing and health

The e-TEORISC contributes to healthcare and advances the nursing discipline by bringing together theory, instruments and technological resources for cardiovascular risk. Furthermore, it can foster the development of robust research and help consolidate advanced nursing practices, fostering autonomy and proactivity among nursing and healthcare professionals. Nurses will be able to lead care for cardiovascular risk and outline direct, precise care aimed at health promotion and prevention.

The e-TEORISC will undergo constant improvements by the research team, and can be used for testing, as a form of assessment, by nursing professionals, and clinical validity with the population at cardiovascular risk. Robust scientific productions should advance in the field of software construction and development, to allow in-depth discussions about the limitations.

Thus, it is postulated that software linked to the needs of identifying cardiovascular risk can, in the medium and long term, contribute to the reduction of cardiovascular risk factors in people, promote health savings and enhance the quality of nursing and healthcare. Once the nursing theory of care in the context of cardiovascular risk is founded, it will be possible to operationalize theoretical components and expand the scope of advanced cardiovascular nursing practice.

## CONCLUSIONS

The e-TEORISC was developed to provide nursing-led care organized by a multidisciplinary team for people at cardiovascular risk, in the form of free, online and offline application software, containing a database linked to NP stages. Experts considered that the attributes of functional suitability, performance efficiency, reliability, maintainability, usability, safety and portability obtained scores higher than 80%, demonstrating the potential for technology transfer to the SUS.
