# Supplementary material for: Software for the care of people with cardiovascular risk: construction and evidence of validity
Source: Rev Bras Enferm. 2024 Dec 16;77(6):e20240276. doi: 10.1590/0034-7167-2024-0276 (PMC11654560; doi:10.1590/0034-7167-2024-0276)
Supplement: Translation

# Translation: *Software* para o cuidado à pessoa com risco cardiovascular: construção e evidências de validade

**DOI:** 10.1590/0034-7167-2024-0276pt

**Authors:** Nuno Damácio de Carvalho Félix, Raércia dos Santos Carneiro, Luis Filippe Rasia Pacheco, Brenda Silva Cunha, João Cruz, Johann Aires Boness, Mariana Carvalho Gavazza, Alba Lúcia Bottura Leite de Barros

**Affiliations:** I Universidade Federal do Recôncavo da Bahia. Santo Antônio de Jesus, Bahia, Brasil; II Universidade Federal do Ceará. Fortaleza, Ceará, Brasil; III Secretaria Municipal de Saúde. Salvador, Bahia, Brasil; IV Universidade Federal de São Paulo. São Paulo, São Paulo, Brasil

**Keywords:** Software, Validação de Programas de Computador, Fatores de Risco de Doenças Cardíacas, Teoria de Enfermagem, Atenção Primária à Saúde

## Abstract

**Objetivos:**

construir e validar um *software* para o cuidado à pessoa com risco cardiovascular.

**Métodos:**

estudo metodológico, aplicado para desenvolvimento de *software*, ancorado em uma teoria e sistema de classificação de enfermagem, em três etapas: 1) engenharia de requisitos; 2) arquitetura de *software* e codificação; e 3) testes e validação de conteúdo por 12 especialistas da área das ciências da computação, com escore da Razão de Validade de Conteúdo.

**Resultados:**

denominada de e-TEORISC, em formato de *software*, para o cuidado de enfermagem a pessoas com risco cardiovascular, *online* e *offline*, contendo um banco de dados vinculados às etapas do Processo de Enfermagem. Os especialistas consideraram que os atributos de adequação funcional, eficiência de desempenho, confiabilidade, manutenibilidade, usabilidade, segurança e portabilidade obtiveram escores desejáveis.

**Conclusões:**

o e-TEORISC possui evidências de validade para instrumentalizar o cuidado à pessoa em risco cardiovascular, com potencial de transferência tecnológica para o Sistema Único de Saúde.

## INTRODUÇÃO

As tecnologias impactam diretamente a vida das pessoas como auxílio às práticas de cuidado e aos modos de viver. O processo de globalização e o rápido desenvolvimento e disponibilidade dos produtos tecnológicos aumentam o acesso às informações e determinam a integração/interação na prestação de serviços de saúde^(1)^, incluindo, nessa seara, o contexto de risco cardiovascular, o qual se tem a demanda de produção técnica e tecnológico para atender à real necessidade de uma população.

O risco cardiovascular compreende um contexto de saúde e de cuidado dependente da identificação de fatores de risco modificáveis e não modificáveis que impactam diretamente a potencialização de condições agudas e/ou crônicas que acometem o sistema circulatório^(2)^, sendo, assim, uma demanda emergente para o cuidado de enfermagem. Logo, faz parte do escopo da ciência de enfermagem desenvolver e avaliar as evidências disponíveis sobre as estratégias de controle e prevenção ao risco cardiovascular por meio de tecnologias. É emergente a necessidade de avanço na produção do conhecimento e fomento de discussões sobre o desenvolvimento de tecnologias com compromisso ético e social e com vistas à resolução de problemas da prática e sustentabilidade no cuidado de enfermagem.

Novas tecnologias têm sido objeto de estudo da enfermagem, com vistas a resolver demandas emergentes do cotidiano, orientadas por uma teoria para transcender o pensar e agir para analisar, interpretar e intervir no processo prático^(3)^. Entre os variados tipos, as tecnologias assistenciais compreendem o resultado da aplicação de métodos e técnicas que elevam as possibilidades de cuidado diante das necessidades da pessoa, com impactos na forma de dispensar cuidados e nas respostas obtidas^(4)^.

No campo da enfermagem, a validação de tecnologias assistenciais tem sido desenvolvida para auxiliar a prática, citando os *softwares*, em especial, como ferramentas inovadoras baseadas em linguagem informatizada que nortearam as intervenções de enfermagem, com objetivos como contemplar um processo interativo e incremental, operacionalizar o sistema logístico-operacional, diminuir o tempo para implementar atividades e facilitar a identificação de demandas de cuidado por meio de reavaliação contínua^(5,6)^. Esses são objetivos essenciais para o desenvolvimento de tecnologias assistenciais com fortes evidências de validade.

Para uso neste estudo, optou-se pela definição de tecnologias assistenciais com foco em evidências de validade, para desenvolvimento de competências clínicas em profissionais de enfermagem e saúde no cuidado ao risco cardiovascular, especialmente na Atenção Primária à Saúde. A tecnologia é um domínio inter-relacionado à enfermagem, com referência ao metaparadigma, e pode proporcionar efeitos positivos na construção de conhecimento^(7)^, com potencial contribuição para a redução dos fatores de risco cardiovascular da população e transferência tecnológica para o Sistema Único de Saúde (SUS).

Há fortes evidências da efetividade de intervenções personalizadas de enfermagem na qualidade de vida de pessoas com doenças cardíacas^(8)^, e os resultados são promissores quando aliam-se o cuidado de enfermagem a essas pessoas e o desenvolvimento de tecnologias assistenciais^(9)^, especialmente quando se tem fundamentação em uma teoria e utiliza um sistema de classificação de enfermagem.

Os sistemas de linguagem padronizada auxiliam a prática do enfermeiro em obter respostas a uma situação clínica de risco ou processo de adoecimento no qual o indivíduo esteja sujeito. Nesse sentido, desponta a carência em verificar as necessidades de diferentes populações por meio do acesso a um plano de cuidados bem estruturado e contendo os diagnósticos de enfermagem, especialmente na atenção primária, cenário ainda carente de processos estruturados e norteados pela classificação de enfermagem^(10)^. Além disso, documentar o Processo de Enfermagem (PE) é uma das prerrogativas éticas da assistência de enfermagem, aumentando a qualidade e a integração de cuidados por meio de cinco etapas bem estabelecidas, inter-relacionadas, recorrentes, interdependentes e cíclicas, mapeando, de forma hierárquica, diferentes modos de uso de sistemas de linguagem^(11)^.

Portanto, faz-se necessário e emergente ao contexto da prática da enfermagem uma tecnologia capaz de auxiliar o enfermeiro a operacionalizar planos de cuidado ao paciente em risco cardiovascular, reduzindo o tempo de consulta, direcionando propostas de cuidado e potencializando indicadores de prática. O *software* construído e avaliado neste estudo se apresenta como original e inovador, ao promover o avanço no conhecimento, aliando a ciência de enfermagem à proposição tecnológica no processo de tomada de decisão liderado por profissionais de enfermagem para o cuidado a pessoas no contexto de risco cardiovascular, em que se tem a escassez de tecnologias como essa no cenário internacional.

## OBJETIVOS

Construir e validar um *software* para o cuidado à pessoa com risco cardiovascular.

## MÉTODOS

### Aspectos éticos

A pesquisa foi aprovada pelo Comitê de Ética em Pesquisa da Universidade Federal do Recôncavo da Bahia, e atendeu aos preceitos éticos da Resolução nº 466 de 2012 do Conselho Nacional de Saúde.

### Tipo do estudo

Trata-se de pesquisa metodológica, aplicada ao desenvolvimento tecnológico^(12)^, do tipo *software*, fundamentada nos pressupostos da Teoria do Cuidado no Contexto de Risco Cardiovascular (TEORISC)^(13)^ e produções associadas ao uso da Classificação Internacional para a Prática de Enfermagem (CIPE®) em relação aos fenômenos de risco cardiovascular e síndrome metabólica^(14-17)^.

### Procedimentos metodológicos

Por meio da técnica de engenharia de *software* ^(18)^, utilizaram-se três etapas de desenvolvimento: 1) engenharia de requisitos; 2) arquitetura de *software* e codificação; e 3) testes e validação de conteúdo por especialistas.

### Etapa 1 - Engenharia de requisitos

Consiste na aplicação de técnicas especializadas para agrupar funcionalidades e descrevê-las com vistas à implementação consistente. As tarefas são revisão e esclarecimento das demandas dos usuários, distribuição, documentação e revisão dos requisitos pelos usuários. Os requisitos foram registrados em dados de usuário para facilitar a comunicação entre time técnico e os membros do Grupo de Pesquisa e Extensão em Cuidado Cardiovascular (GPCARDIO).

Para contemplar essa etapa, realizaram-se cinco fases (SI - *Kick-Off*; SII/III - visão do produto; SIV - o produto é-não é-faz-não faz; SV - objetivos do produto; e SVI - personas). A segunda fase foi dividida em dois momentos (SII e SIII). As reuniões foram conduzidas com ajuda de uma programadora, de forma virtual, utilizando o método *Lean Inception*, a fim de definir o *Minimum Viable Product* (MVP) ou protótipo para, em seguida, construir o *software*.

A SI “*Kick-Off*” consiste na apresentação e explicação do método utilizado e dos seus desenvolvedores. A SII/III “visão do produto” é utilizada para trilhar o caminho inicial do produto. Na SIV, são discutidos os objetivos do produto. A SV correspondeu à fase de desenvolvimento das personas, identificando potenciais usuários da tecnologia através da criação e caracterização de personagens fictícios.

O método *Lean Inception* tem como finalidade acelerar a etapa primária no desenvolvimento do produto e descobrir os pontos mais relevantes para a criação do *software*, tornando-o objetivo. Além disso, promove como resultado o entendimento do MVP, que corresponde à versão mais simples que será apresentada ao usuário. Ainda, o MVP pode direcionar o desenvolvedor sobre a utilidade do *software*, bem como compreender o perfil do usuário e como o produto pode agregar valor e auxiliá-lo^(19)^.

O MVP foi construído para identificar o perfil do usuário que possui o risco cardiovascular, além de pontos mais relevantes para estratégias que determinem soluções para diminuir o risco em adquirir doenças cardiovasculares. Os integrantes do GPCARDIO atuaram nas fases de construção do *software*, na discussão com o engenheiro sobre os requisitos necessários que deveriam conter na tecnologia, conceitos, estrutura, modelagem, sequenciamento de abas e construção de casos hipotéticos.

### Etapa 2 - Arquitetura de *software* e codificação

Compreende a definição de uma estrutura e comportamento de sistema que atenda às expectativas dos seus usuários em termos de eficiência, consistência e segurança, por exemplo. Para elaborar a arquitetura de *software*, foram realizadas três tarefas principais: a) identificação dos atributos de qualidade com descrição dos cenários arquiteturais; b) tomada de decisões arquiteturais; e c) elaboração de visões arquiteturais para o desenvolvimento^(18)^.

Na tarefa A, realizou-se o desenvolvimento das telas, realizado através do *software Web* Canva® com posterior organização, formatação e estruturação pela programadora responsável pelo desenvolvimento do *software*. A arquitetura definida garante a qualidade do sistema e sua documentação, auxiliando os desenvolvedores durante a codificação. Na tarefa B, quanto à segurança das informações do *software*, o acesso aos dados foi permitido apenas a usuários com o número de cadastro específico vinculado à pesquisa, previamente cadastrados pelo administrador - pesquisador proponente do estudo. O *software* foi instalado em um servidor de rede com senhas criptografadas, com o intuito de ampliar a segurança de entrada e registro no sistema.

Na tarefa C, para o desenvolvimento do *software*, foram realizados a codificação e os testes pela programadora. A linguagem de programação utilizada foi a *React com Typescript*, para criação do *frontEnd*. Utilizou-se o SASS para a estilização gráfica, e para a plataforma para hospedagem, foi usado o *Vercel*. Utilizou-se o MSQL como sistema de gerenciamento de banco de dados, que emprega a linguagem *Node.js*, com *TypeScript* como interface, sendo hospedado no servidor em nuvem da empresa *Railway*.

Para o desenvolvimento do *software*, foram utilizados os conceitos “risco cardiovascular”^(2)^ e “síndrome metabólica”^(17)^, uma vez que a síndrome congrega importantes fatores de risco cardiovascular, em que os principais cuidados deverão ser considerados na construção e validação da tecnologia.

Por fim, como ferramenta principal de conteúdo do *software*, utilizou-se um subconjunto terminológico da CIPE®, com 169 intervenções, 55 diagnósticos e 36 resultados de enfermagem, divididos em fatores cardiometabólicos, comportamentais, psicossociais e culturais, laborais, afecciosos, e terapêuticos^(13)^. Ressalta-se que o subconjunto foi validado por 88 especialistas e aplicado a 22 pacientes com síndrome metabólica^(13)^. Assim, operacionalizou-se o instrumento no formato do *software*, maximizando as possibilidades de uso, aplicação e evolução do sistema de classificação.

### Etapa 3 - Testes e validação de conteúdo

Optou-se pela técnica Delphi para teste e validação de conteúdo do *software*, realizado por meio de comitê de *experts* anônimos^(20)^, para análise da representatividade dos itens^(21)^, totalizando 12 especialistas da área das ciências da computação Utilizaram-se como base as normativas da *International Organization for Standardization* (ISO) e da *International Electrotechnical Commision* (IEC), específica para avaliação de *software*.

A primeira etapa do Processo de Avaliação ISO/IEC 25040 é o Modelo de Qualidade de Produto da norma ISO/IEC 25010, em que são avaliadas oito características e 31 subcaracterísticas, direcionadas para especialistas em informática, sendo elas: adequação funcional (integridade funcional, correção funcional e aptidão funcional); eficiência de desempenho (tempo, recursos e capacidade); compatibilidade (coexistência e interoperabilidade); usabilidade (reconhecimento de adequação, apreensibilidade, proteção contra erro, operabilidade, estética da interface do usuário e acessibilidade); confiabilidade (maturidade, tolerância a falhas, recuperabilidade e disponibilidade); segurança (confidencialidade, integridade, não repúdio, responsabilização e autenticação); manutenibilidade (analisabilidade, modificabilidade, modularidade, reusabilidade, testabilidade); e portabilidade (adaptabilidade, capacidade para ser instalado e capacidade para substituir)^(22,23)^.

A validação foi realizada por 12 especialistas, identificados via Plataforma *Lattes*, de acordo com os critérios de inclusão, como ser profissionais das áreas de informática, desenvolvimento de sistemas ou ciências da computação, com experiência na criação de *software* e informatização na saúde ou validação e adaptação de *softwares*. O *expert* que atendesse a todos os requisitos foi incluído na pesquisa. Após contato prévio via correio eletrônico, foram enviados o Termo de Consentimento Livre e Esclarecido, um protocolo de apresentação resumido do projeto de pesquisa, o instrumento adaptado e instruções para preenchimento e avaliação dos quesitos a serem analisados, além de um *link* para acesso ao *software*. Foi estipulado um prazo de 20 dias para a devolução do material analisado.

Utilizou-se a Razão de Validade de Conteúdo (RVC), na qual os especialistas avaliaram cada item do instrumento em +1 (adequado), -1 (adequado com alterações), ou 0 (inadequado). Esses medem a consistência dos itens, sendo considerados relevantes os que obtiverem porcentual mínimo de 80%^(24)^.

### Análise dos dados

Os resultados das duas primeiras etapas foram estruturados e analisados de acordo com as linguagens de programação *Ruby, Ruby on Rails* e *JavaScript*, utilizando-se o *framework Bootstrap* e a implementação de um servidor de produção, em que foram executados *Ubuntu Linux* e *Nginx Webserver*. Os processos de teste e validação de conteúdo por meio do RCV foram estruturados por meio de estatística descritiva.

## RESULTADOS

Na engenharia de requisitos, participou a equipe de pesquisa, composta por cinco discentes de enfermagem, três docentes de enfermagem, pesquisadores enfermeiros e um engenheiro de *software*. Na fase SI, estruturaram-se a apresentação/explicação do método, bem como seus desenvolvedores. A fase SII ajudou a trilhar o caminho inicial do produto, com definições para quem a tecnologia se destina (pessoas adultas), qual problema seria resolvido (cuidado inadequado ao risco cardiovascular), o nome do produto (e-TEORISC) e o que o produto será (*software* de aplicação).

A fase SIII definiu o benefício que o e-TEORISC traz perante o problema (realizar a prevenção de doenças cardiovasculares a médio e longo prazo), a concorrência (clínica tradicional) e o diferencial (e-TEORISC fundamentado em uma teoria e um sistema de classificação de enfermagem). Ressalta-se que o e-TEORISC está direcionado, prioritariamente, às pessoas com fatores de risco (sem doença) cardiovascular. No que tange ao diferencial do e-TEORISC, identificou-se que será capaz de integrar os profissionais de saúde para identificar e assistir, de maneira eficiente e eficaz, o risco cardiovascular das pessoas.

Na fase SIV, entendeu-se que o e-TEORISC é um *software* de aplicação, uma ferramenta para o profissional de saúde liderado por enfermeiros e organizado pela equipe multiprofissional, que funcionará de maneira *online* e *offline*, contendo um banco de dados. Esse banco de dados é onde serão armazenados as informações de cadastro dos pacientes, planos de cuidados e metas compartilhadas entre os profissionais. Definiu-se que e-TEORISC não será uma rede social nem será um assistente automático, *site, checklist* ou protocolo.

Quanto ao que o e-TEORISC faz, definiu-se que o *software* orienta o cuidado ao risco cardiovascular, acompanha parâmetros clínicos e laboratoriais, esclarece dúvidas, compartilha material instrucional, promove o autocuidado, orienta sobre medidas de conforto laboral e avalia a qualidade de vida. Quanto ao que o e-TEORISC não faz, o *software* não determinará diagnósticos/resultados/intervenções ou planos terapêuticos, sendo mantida a autonomia aos profissionais no raciocínio clínico a ser guiado pelo *software*.

Na fase SV, definiram-se como objetivos do e-TEORISC: identificar o risco cardiovascular e a síndrome metabólica; instrumentalizar os profissionais de saúde e de enfermagem para o cuidado no contexto de risco cardiovascular; auxiliar na prescrição de ações para a redução do risco cardiovascular de forma sistematizada; promover a participação ativa da pessoa no cuidado quanto aos fatores de risco cardiovascular; e possuir interoperabilidade para possível transferência tecnológica para o SUS. Na SVI, construíram-se casos de três personagens, tais como enfermeiro, agente comunitário e enfermeiro, definindo suas características sociodemográficas, comportamentais e necessidades em saúde para observar uma testagem do sistema.

Na arquitetura de *software* e codificação, desenvolveu-se a construção do e-TEORISC por meio de uma estrutura da tecnologia dividida em parte inicial com vídeos, logotipo do *software* e-TEORISC e botão de acesso. Os vídeos abordam como usar o *software*, como ele funciona e como acessar os recursos disponíveis (visualização opcional), com informações sobre a equipe de criação da plataforma, com fotos, nomes e treinamento dessas pessoas.

Ressalta-se que a tecnologia é de uso gerencial do enfermeiro e de acompanhamento da equipe de saúde, sendo que o próprio paciente pode fazer seu cadastro para acompanhamento da equipe. Na tela inicial, o botão de acesso mostrará três opções: cliente de saúde (pessoa); profissional de saúde (prestador de cuidado); e desenvolvedor (controlador do *software*). No primeiro acesso, o cliente de saúde realizará o cadastro de forma presencial ou do autopreenchimento, necessitando de nome completo, Cadastro de Pessoa Física, idade, cor, nome social e sexo. Ao final, o cliente deverá manifestar concordância com os termos de uso (Figura 1).

**Figura 1:** Demonstrativo do cadastro da pessoa no e-TEORISC, Santo Antônio de Jesus, Bahia, Brasil, 2023

A consulta presencial inicial deve ser obrigatoriamente com profissional de enfermagem ou de saúde, podendo ser agendada pelo *software*. Serão informados dados clínicos e antropométricos, além de questões sobre prática de exercício físico, sono e repouso, rede de apoio, comorbidades pessoas e/ou familiares e crenças. Para facilitar a distribuição das informações, as telas foram divididas conforme o conjunto de fatores de risco da TEORISC e as informações para preenchimento, rastreamento e estruturação do plano de cuidados por meio do subconjunto da CIPE®.

Através do e-TEORISC, o enfermeiro poderá buscar pelo nome da pessoa e, em seguida, documentar o PE através da realização da avaliação, diagnóstico, planejamento, implementação e evolução de enfermagem, disponibilizando os resultados na aba “registros” e podendo acessar os registros dos demais profissionais, conforme observado nas Figuras 2 e 3.

**Figura 2:** Demonstrativo dos dados referentes ao Processo de Enfermagem no e-TEORISC, Santo Antônio de Jesus, Bahia, Brasil, 2023

**Figura 3:** Demonstrativo dos dados referentes aos fatores de risco cardiovascular no e-TEORISC, Santo Antônio de Jesus, Bahia, Brasil, 2023

Após a primeira consulta, o cliente de saúde poderá ter acesso ao seu histórico de consultas realizadas. Os demais profissionais de saúde poderão participar de interconsultas (se necessário), com agendamento de dia e horário. Os registros serão feitos de acordo com o plano de cuidados individualizado de cada profissional, tendo acesso a outros registros profissionais e dados dos clientes. Os desenvolvedores terão acesso a todas as informações da plataforma, incluindo o banco de dados (em nuvem) após o cadastramento.

Os especialistas avaliadores possuem formação em ciência da computação (100%), sendo formados por doutores (40%), mestres (50%) e especialistas (10%), com atuação profissional predominante na docência (60%), seguidos de atividades autônomas (40%) ligadas ao desenvolvimento e avaliação de produtos tecnológicos do tipo *software* para serviços de saúde. Nos testes e validação de conteúdo, o e-TEORISC obteve a média de 89,9% da RVC, considerando válido o conteúdo, conforme a Tabela 1. Entre os atributos, destaca-se a portabilidade, com RVC de 100%. Destaca-se que os atributos usabilidade, segurança e portabilidade obtiveram escores acima de 90%. Os atributos adequação funcional, eficiência de desempenho, confiabilidade e manutenibilidade apresentaram subcaracterísticas com porcentuais de 80% a 85%.

**Tabela 1:** Porcentual de concordância dos especialistas em relação à Razão de Validade de Conteúdo de cada atributo e subcaracterísticas em relação ao *software* e-TEORISC, Santo Antônio de Jesus, Bahia, Brasil, 2023

Atributos	Subcaracterísticas	RVC (%)^*^
Adequação funcional	Integridade funcional	85
	Correção funcional	85
	Aptidão funcional	85
Eficiência de desempenho	Comportamento do tempo	80
	Uso dos recursos	85
	Capacidade	85
Compatibilidade	Coexistência	75
	Interoperabilidade	75
Usabilidade	Reconhecimento de adequação	95
	Apreensibilidade	100
	Proteção contra erro	95
	Operabilidade	95
	Estética da interface do usuário	100
	Acessibilidade	95
Confiabilidade	Maturidade	75
	Tolerância a falhas	85
	Recuperabilidade	85
	Disponibilidade	85
Segurança	Confidencialidade	95
	Integridade	90
	Ausência de repúdio	90
	Responsabilização	90
	Autenticação	100
Manutenibilidade	Analisabilidade	85
	Modificabilidade	85
	Modularidade	80
	Reusabilidade	80
	Testabilidade	85
Portabilidade	Adaptabilidade	100
	Capacidade para ser instalado	100
	Capacidade para substituir	100

* *RVC - Razão de Validade de Conteúdo.*

As subcategorias coexistência, interoperabilidade e maturidade obtiveram RVC abaixo do estabelecido (75%), em que os especialistas julgaram que o e-TEORISC precisa avançar no que se refere aos *softwares* já existentes no SUS, assim como a testagem clínica, para fortalecimento dos indicadores relacionados aos itens. As principais considerações são apresentadas a seguir: melhorar a estrutura da diagramação das ferramentas relacionadas aos itens da consulta de enfermagem; aprimorar as funções de forma mais eficiente enquanto compartilha um ambiente ou recursos com outros produtos; melhorar a capacidade do *software* em possibilitar que dois ou mais sistemas, produtos ou componentes possam trocar informações; e aprimorar a capacidade do *software* fortalecer a confiabilidade, por meio de avaliação por profissionais da atenção primária e testes clínicos robustos, retroalimentando e amadurecendo o e-TEORISC. Por se tratar de uma tecnologia vinculada institucionalmente ao patrimônio da universidade em que foi desenvolvida, o *software* passará por registro no Instituto Nacional da Propriedade Industrial e, após a concessão da marca à universidade, poderá ser disponibilizado para transferência tecnológica ao SUS.

## DISCUSSÃO

O e-TEORISC foi idealizado com base no PE e fundamentado na TEORISC, que prevê o fenômeno do risco cardiovascular e auxilia nas práticas de cuidado com a promoção da saúde, o que permeia novo grau de empoderamento aos enfermeiros da atenção primária na contenção de agravos e prevenção de doenças, além de permitir a geração de indicadores baseados em linguagem especializada de enfermagem. Seu uso permite aspirar um programa de cuidados a pessoas com risco cardiovascular, pela capacidade de triagem, identificação de riscos, propostas de intervenção e auxílio ao raciocínio clínico de enfermagem, especificamente a saúde cardiovascular.

Em pesquisa que implementou o PE em unidades de internamento hospitalar, foi previsto que o uso de *software* pode permitir tomada de decisão, julgamento clínico, prover diagnósticos, resultados e intervenções de enfermagem em *modus operandi* e diretivo^(25)^. Contudo, a continuidade dos cuidados e do uso do sistema não foi plenamente atingido, o que leva a fragilidades quanto à transferência tecnológica para o serviço de saúde. Esse é um ponto crucial de operação do e-TEORISC, ao possibilitar agilidade no PE e assegurar a efetiva aplicabilidade nos serviços, podendo demonstrar rápida absorção pelos enfermeiros do serviço.

No campo da saúde cardiovascular, existem programas de *software* que respondem às necessidades de verificação das curvas do eletrocardiograma como auxílio na identificação de anomalias relacionadas com patologias cardíacas, bem como ao conceito de insuficiência cardíaca, causas, sinais e sintomas, diagnóstico e tratamento^(26,27)^. No campo da assistência de enfermagem, não foram encontrados *softwares* que amparem os enfermeiros na condução de planos de cuidados a pacientes com risco cardiovascular, o que se constitui uma importante lacuna no cenário internacional. No e-TEORISC, é permitido ao enfermeiro a digitação de respostas observadas não contempladas no esquema de telas apresentado, o que avança significativamente no desenvolvimento do *software* e não prende a um escopo padrão, possibilitando sua remodelação contínua.

No que concerne à apresentação em tela, observa-se a produção de um *software* viável, com estruturas típicas da arquitetura e linguagem de máquinas (*mechanic learning*), o que congrega cinco fases bem delimitadas e que viabilizam a operacionalização do sistema. Contudo, devido ao ineditismo de uma produção tecnológica no âmbito do risco cardiovascular, é importante delimitar sua necessidade de adequação ao ambiente digital, bem como atualizações frequentes próprias de pesquisas com esse escopo.

O e-TEORISC foi dividido nos grupos de fatores que compõem a teoria que fundamentou este estudo, por isso se preocupou em agrupar todos os indicadores pertencentes ao risco cardiovascular com diagnósticos e intervenções agrupadas e segmentadas a cada grupo de fatores. Entende-se que um sistema que contemple a maior quantidade de elementos capazes de identificar um fenômeno de enfermagem assegura melhores resultados à dinâmica profissional^(28)^. Nessa perspectiva, a contribuição da TEORISC para este estudo envolve diferentes vertentes, com enfoque nas etapas do PE pelo instrumento construído e validado^(13)^, como base a compreensão das etapas do PE com a prevenção de doenças cardiovasculares, melhorando assimilações clínicas dos profissionais de enfermagem.

O *software* permite a identificação dos fatores de risco cardiovascular, traçando alternativas de cuidado a partir da tomada de decisão clínica. Em contraponto, a literatura descreve a utilização do *software* REC24h, que faz parte do Estudo de Riscos Cardiovasculares em Adolescentes, porém limitando-se aos fatores da obesidade, das alterações no metabolismo lipídico e glicêmico e da pressão arterial^(29)^.

Os *softwares*, de forma geral, já fazem parte da rotina dos serviços de saúde, com vistas a ampliação de um método holístico, acessível e amplamente usado. Em alguns casos, ajudam a operacionalizar serviços com a finalidade de congregar um banco de dados para gerar relatórios, fluxos e dicionários^(30)^. Nesse caso, o *software* em questão utiliza a CIPE® como sistema de classificação. Além de 55 diagnósticos, 169 intervenções e 36 resultados de enfermagem para o público em risco cardiovascular, os enfermeiros podem optar por quais diagnósticos e intervenções desejam aplicar no indivíduo, a partir do agrupamento de dados e da tomada de decisão. Quanto a isso, estudo aponta a necessidade de inserir linguagens padronizadas nas tecnologias de cuidado que promovam a orientação para o PE, por meio do suporte à tomada de decisão, de forma que seja acessível tanto ao profissional quanto ao usuário^(25)^. Essa prerrogativa foi contemplada no estudo em tela.

O uso do *software* implementa linguagens lógicas e padronizadas, na tentativa de estruturar planos de cuidado para o risco cardiovascular. Assim, *softwares* com essa temática podem corroborar o rastreamento do risco cardiovascular, e tendem a incentivar o autocuidado com a saúde quando esse risco é identificado. Como consequência, tem-se a redução da morbimortalidade por causas cardiovasculares^(31)^. Por sua vez, deve-se atentar para as estratégias já implementadas ao risco de desenvolver doenças cardíacas para aprimorar estratégias de cuidado.

Os *softwares* são meios que possibilitam otimizar os registros de enfermagem e, assim, dinamizar suas ações. Dessa forma, eles auxiliam na tomada de decisão, ao combinar diferentes fontes de saber, como a engenharia de *software* e o raciocínio clínico, formulando perfis adequados de usuários^(25)^, registros diretos e objetivos das informações de saúde em diferentes contextos^(26)^, liberdade, padronização, diminuição de tempo em registros burocráticos e resgate facilitado de informações^(32)^, além da necessidade de reavaliação permanente^(33)^. Ressalta-se que o e-TEORISC em tela tem a finalidade de corroborar os objetivos expostos na literatura, bem como o armazenamento sistemático da informação.

Os dispositivos difundidos na literatura científica não predizem ou norteiam o cuidado, mas concentram-se na identificação do risco. O HEARTS é uma iniciativa da Organização Mundial da Saúde com alvo na Atenção Primária à Saúde que visa melhorar a prevenção e o controle de infarto do miocárdio, acidente vascular cerebral ou óbito cardiovascular ao longo de dez anos^(34)^. Outro aplicativo é o ASCVD *Risk Estimator*, estimando o risco cardiovascular para os próximos dez anos. Também não se trata de um aplicativo que pode guiar uma consulta, mas que funciona com um rastreador para alguns fatores de risco, salientando que o risco de desenvolver doenças cardiovasculares só pode ser calculado considerando a faixa etária de 40 a 79 anos^(35)^. Apesar de serem ferramentas importantes, não incluem diversos fatores de riscos necessários para uma avaliação mais completa da saúde da pessoa com risco cardiovascular.

Em programas de cuidado liderados por enfermeiros em saúde cardiovascular, as intervenções baseiam-se no uso de teorias de enfermagem que permitam aumentar a resiliência em pacientes após angioplastia coronariana^(36)^, ou na diminuição de fatores de risco após um evento agudo, como as síndromes coronarianas^(37)^. Nesse caso, é essencial que os enfermeiros avaliem o e-TEORISC e proponham alternativas para aperfeiçoar o sistema, visando sua liderança nos cuidados cardiovasculares e adequação às suas necessidades e distintas realidades.

Estudos com validação de *softwares* na enfermagem observaram a proporção de validade similar à encontrada no estudo em tela, como no *software* para insuficiência cardíaca, com validação maior que 0,80 em seus itens^(26)^, e *software* para o cuidado em hemodiálise (0,89)^(38)^, o que corrobora o estudo em tela. A maior parte das categorias avaliadas neste estudo recebeu RVC maior que 0,80, exceto a coexistência, interoperabilidade e maturidade, apontando a demanda de seguimento no desenvolvimento e avaliação clínica do e-TEORISC. Esses necessitam de revisão, assim como em outros estudos que alcançaram escores menores de 0,78^(38)^. Por outro lado, estudo com *software* para prevenção de lesões em recém-nascidos, utilizando a mesma normativa ISO/IEC 25040, adotou como válidas as definições acima de 0,70^(39)^. Essa alternativa dá subsídios para revisões leves nos julgamentos que obtiveram escore menor que 0,75 no estudo em tela.

Fatores como estresse laboral e familiar, sedentarismo e antecedentes familiares estão ausentes em outras tecnologias^(32)^, sendo mais um diferencial para a tecnologia assistencial fruto deste estudo, que é embasada em uma teoria de enfermagem na qual o próprio conceito de risco cardiovascular foi analisado, dando robustez ao *software*. Estudos similares ainda confirmam a ideia de que as tecnologias assistenciais, como *softwares*, contribuem para tornar o serviço de saúde mais ágil nas etapas de coleta, registro, armazenamento e manipulação dos dados dos pacientes, o que ajuda a diminuir possíveis erros no processo de cuidado^(33)^.

A construção deste produto tecnológico, alicerçado na TEORISC, proporciona suporte teórico ao *software*, no sentido de trazer elementos que possibilitam contribuir com o pensamento crítico do enfermeiro na operacionalização do PE^(11)^. Esse processo culmina na translação do conhecimento teórico em prática. Ainda, destaca-se a necessidade de atenção especial aos fatores de risco cardiovascular, já que esses são múltiplos e contribuem para a elevação do risco de desenvolvimento de doenças cardiovasculares, assim como a elevação da morbimortalidade, havendo necessidade de reconhecimento desses fatores no processo de desenvolvimento do cuidado^(2)^.

Dessa forma, a TEORISC, associada ao *software* desenvolvido, configura-se instrumento valioso para a prática de enfermagem, pois orienta o cuidado de saúde sobre a ótica do enfermeiro, embasado cientificamente e utilizando um sistema de linguagem padronizada, contribuindo também para a difusão da importância do uso de sistemas de classificação em enfermagem e da incorporação do PE na prática cotidiana que, por sua vez, pode gerar dados e indicadores para os serviços de saúde.

### Limitações do estudo

As sugestões dos experts ainda não puderam ser incorporadas plenamente ao e-TEORISC devido aos trâmites necessários às modificações do sistema. O *software* precisa de aprimoramentos para sua incorporação ao SUS com toda sua função operante. A participação de integrantes com expertise na área e o seguimento do rigor metodológico foram seguidos para reduzir as limitações.

### Contribuições para as áreas da enfermagem e saúde

O e-TEORISC contribui no cuidado de saúde e avança no escopo da disciplina de enfermagem ao congregar teoria, instrumentos e recursos tecnológicos ao risco cardiovascular. Ademais, poderá fomentar o desenvolvimento de pesquisas robustas e ajudar na consolidação de práticas avançadas de enfermagem, fomentando autonomia e proatividade aos profissionais de enfermagem e saúde. O enfermeiro poderá liderar os cuidados ao risco cardiovascular traçar cuidados diretos, precisos e com vistas à promoção e prevenção à saúde.

O e-TEORISC passará por constantes aprimoramentos realizados pela equipe de pesquisadores, e poderá ser utilizado para testagem, como forma de avaliação, por profissionais de enfermagem, e validação clínica com a população com risco cardiovascular. As produções científicas robustas devem avançar no campo da construção e desenvolvimento do *software*, para permitir discussões aprofundadas sobre os limitantes.

Assim, postula-se que um *software* atrelado às necessidades de identificar o risco cardiovascular pode, a médio e longo prazo, contribuir na redução dos fatores de risco cardiovascular das pessoas, promover economia em saúde e potencializar a qualidade dos cuidados de enfermagem e saúde. Uma vez fundamentada a teoria de enfermagem do cuidado no contexto do risco cardiovascular, será possível operacionalizar componentes teóricos e ampliar o escopo da prática avançada de enfermagem cardiovascular.

## CONCLUSÕES

Desenvolveu-se o e-TEORISC para instrumentalizar o cuidado liderado por enfermeiros e organizado em equipe multiprofissional para a pessoa em risco cardiovascular, em formato de *software* de aplicação gratuito, *online* e *offline*, contendo um banco de dados vinculado às etapas do PE. Os especialistas consideraram que os atributos de adequação funcional, eficiência de desempenho, confiabilidade, manutenibilidade, usabilidade, segurança e portabilidade obtiveram pontuação maior que 80%, demonstrando a potencialidade de transferência tecnológica para o SUS.
